# Immunoproteomic mediators of diabetic peripheral neuropathy: causal insights from Mendelian randomization and single-cell validation

**DOI:** 10.3389/fimmu.2026.1681223

**Published:** 2026-01-29

**Authors:** Chenhao Hu, Shengqiao Sun, Dezhi Li, Lebao Yu, Chao Guo, Song Liu

**Affiliations:** 1 Beijing Key Laboratory of Central Nervous System Injury, Beijing Neurosurgical Institute, Capital Medical University, Beijing, China; 2 Department of Neurosurgery, Beijing Tiantan Hospital, Capital Medical University, Beijing, China; 3 U1195, Inserm et Universite Paris-Saclay, Le Kremlin-Bicetre, France

**Keywords:** diabetic peripheral neuropathy, DPN, Mendelian randomization, MR, single-cell

## Abstract

**Objective:**

To elucidate causal roles of circulating immune cells and plasma proteins in diabetic peripheral neuropathy (DPN) pathogenesis using integrative Mendelian randomization (MR) and single-cell validation.

**Methods:**

We employed two-sample MR to assess causal effects of 731 immune traits and 4,719/2,923 plasma proteins on DPN risk (FinnGen: 2,843 cases/389,580 controls). Significant exposures underwent mediation MR to identify protein-immune interactions. Validation included scRNA-seq of sural nerves (4 DPN vs. 3 controls) and flow cytometry/immunofluorescence for dendritic cell infiltration.

**Results:**

Five HLA-DR^+^ immune phenotypes (predominantly dendritic subsets) increased DPN risk (OR = 1.27–1.63, FDR<0.05). Six plasma proteins conferred protection (MICB, HLA-DRA, CAPS, CD79B, AGER, PRKCG; OR = 0.10–0.58). Mediation MR revealed pathogenic immune cells suppress CAPS/HLA-DRA expression (mediating 26.3% of neurotoxicity, P<0.001), while MICB attenuated DPN by inhibiting immune phenotypes. scRNA-seq confirmed conventional dendritic cell infiltration in DPN nerves and disrupted reparative crosstalk with Schwann cells.

**Conclusion:**

We establish a causal neuroimmune network wherein HLA-DR^+^ dendritic cells drive DPN through suppression of protective plasma proteins (CAPS/HLA-DRA), while MICB exerts dual protection. These findings reveal novel therapeutic targets for DPN intervention.

## Introduction

Diabetic peripheral neuropathy (DPN), characterized by damage to nerves within the peripheral nervous system, is a complex, frequently occurring, and incapacitating disorder. Epidemiological studies indicate that it affects more than 8.5% of the general population (1), with the prevalence increasing to nearly 20% among patients with diabetes (2). At present, glycemic management and pain alleviation remain the primary effective interventions. Although intensive glycemic control significantly reduces the incidence of neuropathy in individuals with type 1 diabetes (T1D), its preventive efficacy appears to be substantially diminished in those with type 2 diabetes (T2D) (3). The complex metabolic and intracellular signaling pathways underlying DPN pathogenesis remain incompletely understood, necessitating deeper mechanistic insights and the development of novel, safe, and effective therapeutics.

Accumulating evidence implicates immune dysregulation in DPN, which is characterized by inflammatory cell infiltration in peripheral nerves (4). Comparative analyses further revealed altered immune cell profiles—notably, increased numbers of M0/M2 macrophages, neutrophils, and follicular helper T cells—in DPN patients compared with healthy controls (5). Critically, activated immune cells secrete key proinflammatory cytokines (e.g., TNF-α, IL-1β, IL-6, and IL-8), propagating inflammatory cascades that drive nerve damage (6). Nevertheless, fundamental questions persist regarding the causal roles of specific immune mediators (e.g., dendritic cells) in DPN pathogenesis. Conventional observational studies also face inherent limitations, including confounding, reverse causation, and selection bias, that obscure a causal inference. Mendelian randomization (MR) overcomes these constraints by leveraging genetic variants as instrumental variables, effectively simulating randomized controlled trials to establish robust causal relationships.

In addition, many plasma proteins act as principal regulators of molecular pathways associated with diabetes complications (7). Memarian E showed that plasma protein N-glycosylation is associated with cardiovascular disease, nephropathy, and retinopathy in patients with type 2 diabetes (8). Proteome-wide MR analyses identified 21 blood proteins that may be causally associated with diabetic kidney disease (9). Recent evidence also implicates immune cells in type 2 diabetes pathology, as evidenced by elevated circulating levels of acute-phase proteins (including C-reactive protein (CRP), haptoglobin, fibrinogen, plasminogen activator inhibitor, and serum amyloid A), sialic acid, cytokines, and chemokines (10). Therefore, elucidating the causal interplay between circulating immune cells and plasma proteins and their collective impacts on DPN through MR is crucial for identifying novel therapeutic targets.

We applied a two-sample MR framework to address these gaps, and systematically assessed 731 immune cell phenotypes and 4,719/2,923 plasma proteins as exposures against DPN outcomes. Our MR analyses identified five causal immune cell phenotypes (predominantly HLA-DR^+^ dendritic subsets) and six protective plasma proteins (e.g., MICB and HLA-DRA) associated with DPN. Crucially, the mediation MR analysis revealed that elevated immune cell numbers reduce CAPS and HLA-DRA expression, thereby increasing the DPN risk, whereas MICB attenuates DPN by suppressing pathogenic immune cells. Single-cell validation further confirmed conventional dendritic cell infiltration in DPN nerves and impaired reparative crosstalk with Schwann cells. This integrative approach establishes a causal immune–plasma protein network that drives DPN pathogenesis.

## Methods

### Study design

**Figure 1:**
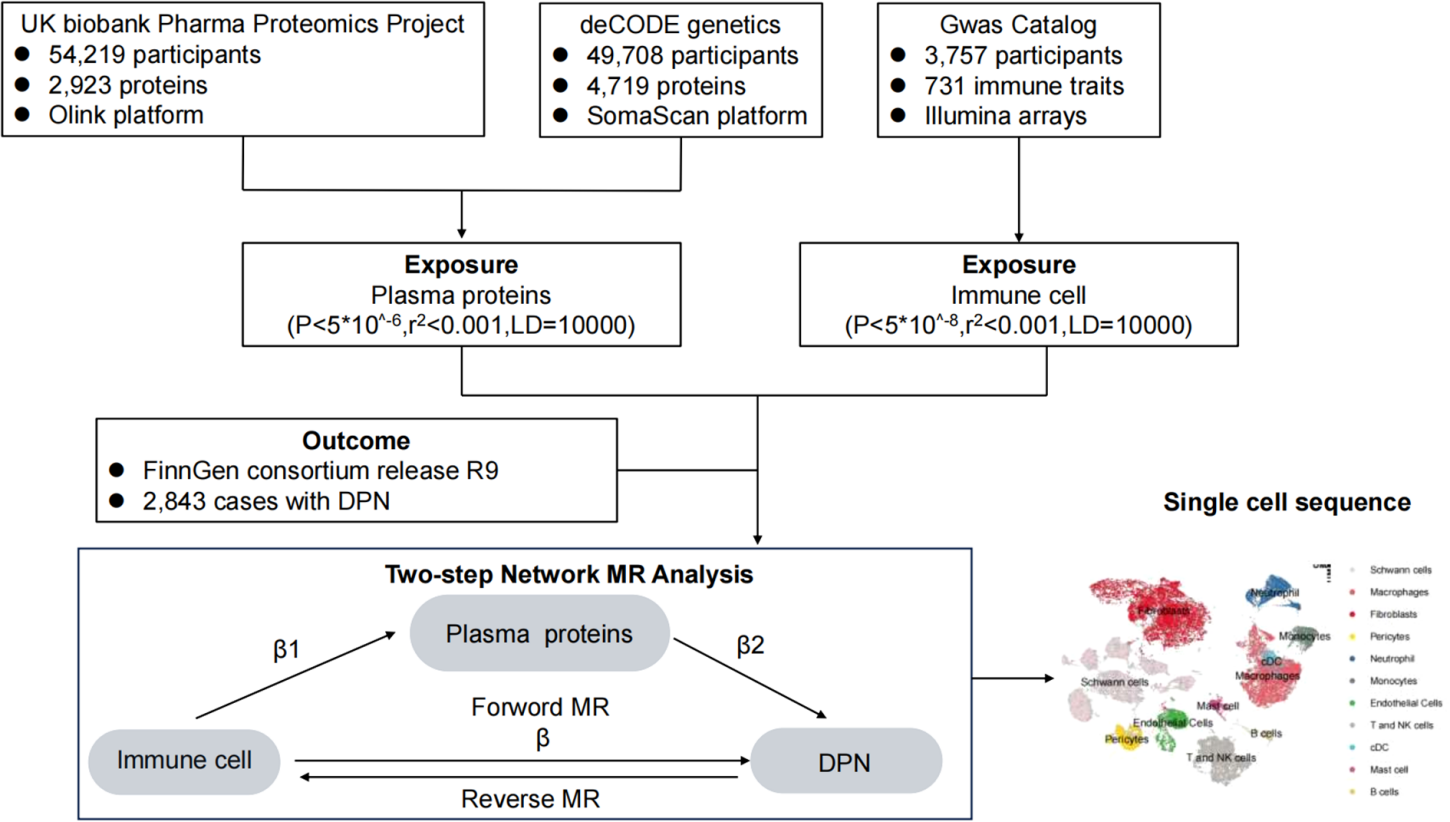
Flowchart of this study.

We employed a two-step MR framework to first estimate the causal effects of genetically predicted levels of circulating immune cell traits and plasma proteins on the DPN risk using two-sample MR, identifying significant exposures for further analysis; subsequently, we assessed the causal effects of these significant immune cell traits on the significant plasma proteins and calculated the proportion of the causal effect of each immune cell trait on DPN mediated by each plasma protein, formally quantifying the indirect effect via the mediator. MR leverages genetic variants as instrumental variables (IVs) to infer causality, requiring these IVs to satisfy three core assumptions: (1) a direct association with the exposure; (2) no association with confounders (known or unknown) of the exposure–outcome relationship; and (3) no influence on the outcome except through the exposure pathway. All included studies received approval from relevant institutional review boards, with participants providing informed consent. The overall analytical approach is depicted in **Figure 1**.

### Description of the data sources

Plasma proteomic and genetic data were sourced from two studies: deCODE Genetics (49,708 Icelandic individuals; 4,719 plasma proteins) and the UK Biobank Pharma Proteomics Project (UKB-PPP) (54,219 participants; 2,923 plasma proteins). Genome-wide association study (GWAS) summary statistics for 731 immune traits were sourced from the GWAS Catalog (accessions GCST0001391–GCST0002121). The original analysis utilized nonoverlapping data from 3,757 European individuals. It comprised absolute cell counts (ACs, n=118), median fluorescence intensities (MFIs, n=389; surface antigen levels), morphological parameters (MPs, n=32), and relative cell counts (RCs, n=192). These immunophenotypes included B cells, dendritic cells (DCs), mature T cells, monocytes, myeloid cells, and TBNK/Treg panels (MFI/AC/RC), whereas the MP features covered DC and TBNK panels exclusively. The GWAS summary statistics for DPN were sourced from the FinnGen consortium release R9. Additionally, the GWAS summary data for DPN included 2,843 patients and 389,580 controls.

### Genetic instrumental variable selection

For the selection of genetic instruments, plasma proteins and DPN were initially screened using a genome-wide significance threshold of P<5×10^-8^. Due to the limited number of eligible SNPs identified under this stringent criterion, the threshold was relaxed to P< 1×10^-6^ to ensure an adequate number of instrumental variables. In contrast, SNPs for immune cell traits were selected strictly based on genome-wide significance (P<5×10^-8^). To ensure independence among instruments and avoid bias from linkage disequilibrium (LD), we applied clumping using the clump_data function from the TwoSampleMR package (version 0.6.22) in R, removing SNPs in LD (r^2^>0.001) within a 10,000 kb window. Finally, to assess instrument strength and mitigate weak instrument bias, we calculated the F-statistic for each SNP and retained only those with F>10.

### MR analysis

We used the default parameters of the function harmonize_data (exposure_dat, outcome_dat, action = 2) from the TwoSampleMR package to harmonize the data, ensuring consistent alignment of the effect direction for each specific allele on both the exposure and the outcome. Primary causal estimation employed the Wald ratio for single-instrument analyses, whereas the inverse variance weighted (IVW) and MR–Egger methods were utilized for analyses with ≥2 instruments. Sensitivity analyses included Cochran’s Q statistic (heterogeneity assessment), MR–Egger intercept testing (horizontal pleiotropy detection), and leave-one-out analysis (influential variant identification). All inferences incorporated false discovery rate (FDR) correction via the Benjamini–Hochberg procedure, with an FDR-adjusted P < 0.05 considered significant for two-sided tests, implemented using the TwoSampleMR package in R.

### Single-cell RNA sequencing data analysis

scRNA-seq data for diabetic peripheral neuropathy (DPN; GSE266026), comprising sural nerve samples from 4 DPN patients and 3 traumatic limb amputation (TLA) controls, were obtained from the GEO database. This publicly available dataset possessed all the requisite ethical approvals. The raw 10x Chromium data were processed using CellRanger (v4.0.10) for GRCh38-aligned read quantification, followed by sparse matrix conversion via Seurat (v4.3.0). Cells expressing <500 genes or exhibiting >10% mitochondrial reads were excluded. Doublets were identified and removed using DoubletFinder (v2.0.3) with sample-specific parameter optimization. After SCTransform normalization, batch correction was performed with Harmony (v1.2.0). The top 40 Harmony dimensions informed the UMAP visualization. Principal components for downstream analyses were selected using elbow plots and a heatmap inspection of the variance distribution, with the clustering resolution set to 0.8 using Seurat’s FindNeighbors/FindClusters functions. The differential gene expression analysis was performed with the FindAllMarkers function. We examined potential intercellular communication with the CellChat R package, version 1.6.1.

### Flow cytometry and immunofluorescence analyses of dendritic cell marker expression

Dendritic cells were quantified in blood and nerve tissues from 3 healthy controls and 3 DPN patients using CD11c as a conserved marker to validate the scRNA-seq findings. For peripheral blood, flow cytometry (BD FACSLyric™) with anti-CD11c-FITC (BioLegend #301629) was used to establish absolute DC counts in EDTA-anticoagulated samples, including isotype controls and technical triplicates. Sections of nerve tissue were subjected to immunofluorescence staining using an anti-CD11c antibody (Abcam ab52632; 1:100), an Alexa Fluor 594-conjugated secondary antibody, and DAPI counterstaining. Confocal imaging (Zeiss LSM 900) followed by ImageJ analysis were used to quantify the CD11c^+^ areas. All experiments included triplicate measurements of each sample.

## Results

### Identification of the causal effects of circulating immune cells on DPN

**Figure 2:**
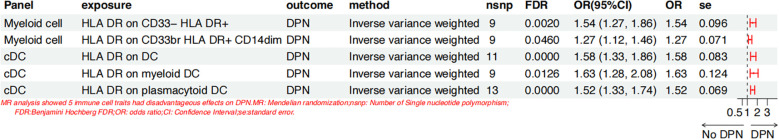
Causal associations between circulating immune cells and DPN.

We identified five distinct immune cell types that exhibited nominally significant causal effects on diabetic peripheral neuropathy (DPN), with elevated levels associated with an increased disease risk. These cell types included specific myeloid subsets (HLA DR on CD33^-^ HLA DR+ [OR = 1.54; 95% CI = 1.27–1.86; FDR = 0.0020] and HLA-DR+CD33brCD14dim [OR = 1.27; 1.12–1.46; FDR = 0.0460]) and conventional dendritic cell populations (HLA-DR+ DCs [OR = 1.58; 1.33–1.86; FDR<0.001], HLA-DR+ myeloid DCs [OR = 1.63; 1.28–2.08; FDR = 0.0126], and HLA-DR+ plasmacytoid DCs [OR = 1.52; 1.33–1.74; FDR<0.001]) (**Figure 2**). No evidence of heterogeneity was observed for these causal relationships (all P > 0.05, **Supplementary Table 1**). Sensitivity analyses, including leave-one-out validation, confirmed the robustness of these associations, as removing individual instrumental variables did not substantially alter the overall causal estimates (**Supplementary Figure 1**). Reverse-direction Mendelian randomization analysis confirmed that DPN has no causal effect on immune cells (**Supplementary Figure 2**).

### Identifying the causal effects of plasma protein levels on DPN

**Figure 3:**
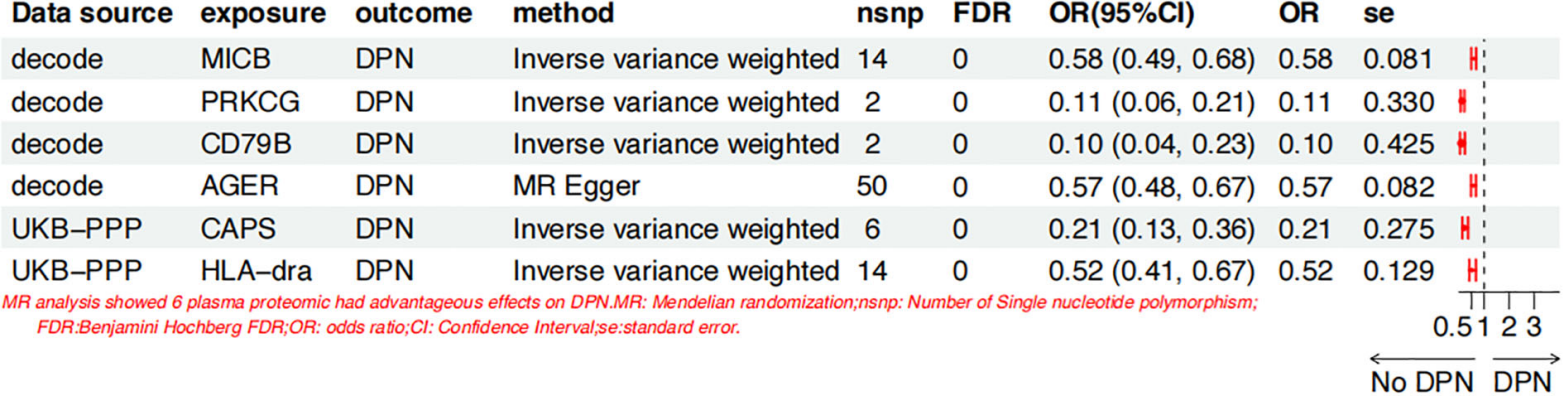
Causal associations plasma proteins and DPN.

Utilizing plasma protein data from 35,559 Icelanders in the deCODE Genetics study and 54,219 participants in the UK Biobank (UKB), we performed two-sample MR (MR) analyses to assess the causal associations of 4,719 and 2,923 plasma proteins with DPN, respectively. The MR analyses revealed that the levels of six unique plasma proteins exhibited significant causal associations with DPN (false discovery rate (FDR) < 0.05). Four proteins were detected in the deCODE cohort, and two were detected in the UKB cohort. Genetically predicted increases in the levels of these six proteins were associated with a decreased risk of DPN: major histocompatibility complex class I polypeptide-related sequence B (MICB) [odds ratio (OR) = 0.58, 95% confidence interval (CI): 0.49–0.68, FDR < 0.05]; protein kinase C gamma (PRKCG) [OR = 0.11, 95% CI: 0.06–0.21, FDR < 0.05]; CD79b molecule (CD79B) [OR = 0.10, 95% CI: 0.04–0.23, FDR < 0.05]; advanced glycosylation end-product specific receptor (AGER) [OR = 0.57, 95% CI: 0.48–0.67, FDR < 0.05]; calcyphosine (CAPS) [OR = 0.21, 95% CI: 0.13–0.36, FDR < 0.05]; and major histocompatibility complex, class II, DR alpha (HLA-DRA) [OR = 0.52, 95% CI: 0.41–0.67, FDR < 0.05] (**Figure 3**). Importantly, the 6 identified causal proteins exhibited consistent directional estimates in all MR methods (MR–Egger, weighted median, weighted mode) compared with the IVW estimates, which confirmed that our MR findings were highly reliable (p value< 0.05) (**Supplementary Table 2**). When the 6 plasma proteins were considered as exposure factors and DPN as the outcome, an MR analysis was conducted along with an MR-PRESSO test (p > 0.05), indicating no pleiotropy or unbiased SNPs (**Supplementary Table 3**). This result led to the determination of the effect size β2 from plasma proteins to DPN.

### Mediation MR analysis

**Figure 4:**
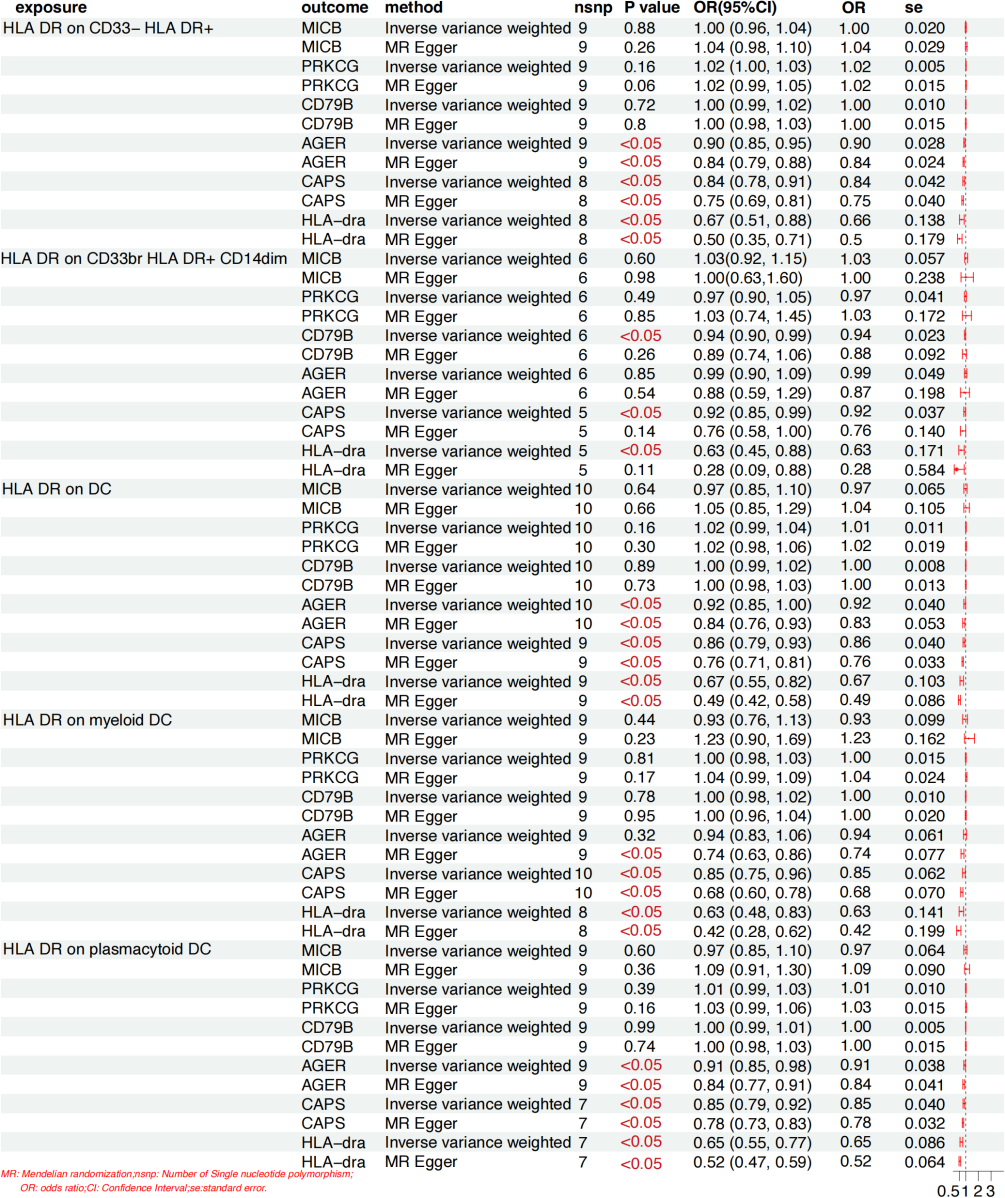
Mendelian randomization estimation of the association between positive circulating immune cells and proteins. Immune cells were treated as exposures and plasma proteins as outcomes.

**Figure 5:**
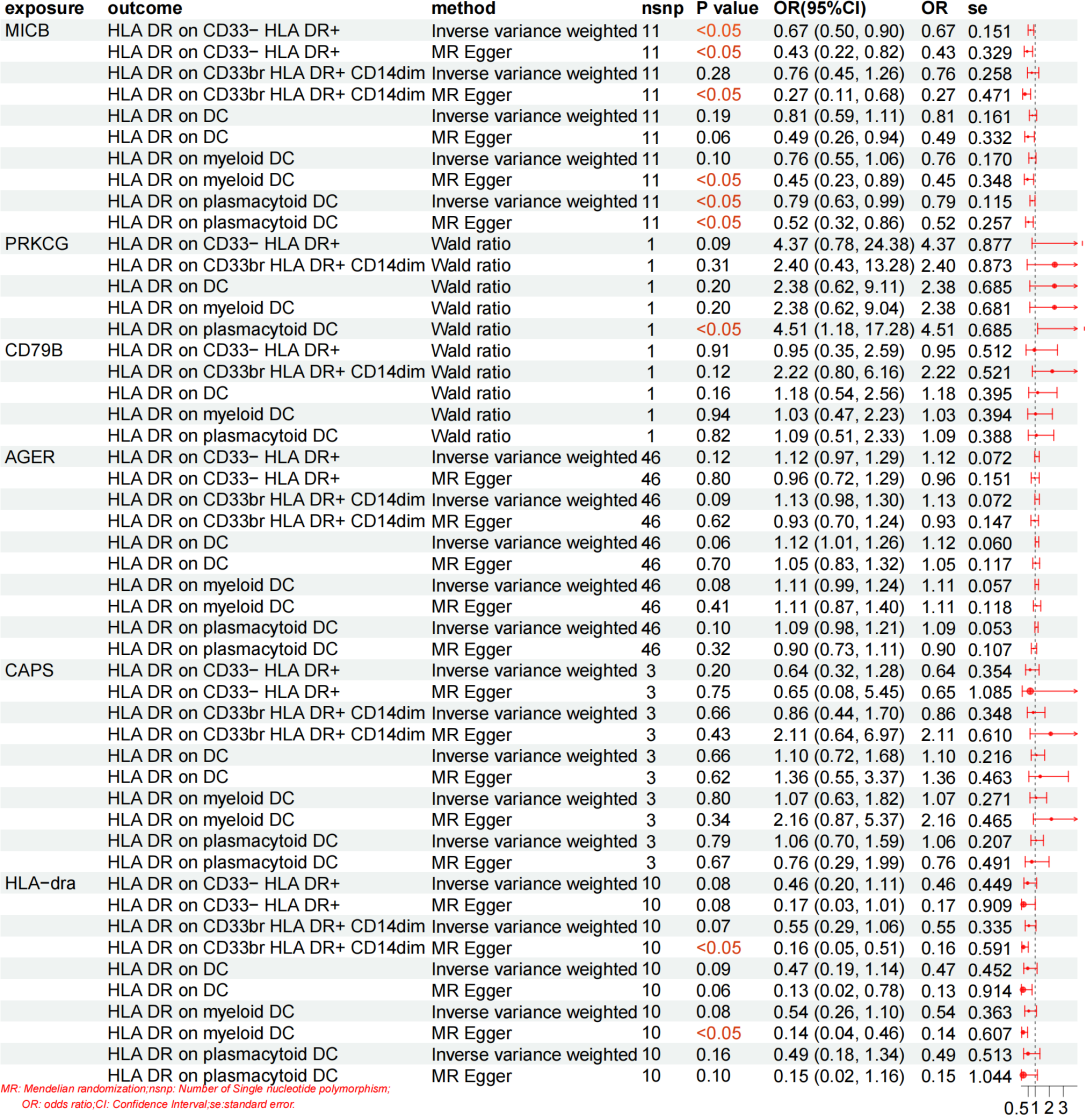
Mendelian randomization estimation of the association between positive circulating immune cells and proteins. Plasma proteins were treated as exposures and immune cells as outcomes.

Building upon the previously identified immune cells and plasma proteins, we employed MR to further compute the mediation effect. Using five selected immune cell phenotypes as exposures and six plasma proteins as outcomes, we performed an MR analysis to assess the causal effects of immune cells on plasma proteins. This analysis revealed causal relationships between the presence of five immune cell phenotypes and the levels of four plasma proteins (CD79B, AGER, CAPS, and HLA-DRA), and the effect sizes for these pathways were quantified. The analysis revealed that a single immune cell phenotype could have causal relationships with multiple proteins. For example, the presence of HLA DR on the CD33− HLA DR+ subset not only was negatively correlated with AGER levels but also with CAPS levels. Similarly, the presence of HLA-DR on the CD33br HLA-DR+CD14dim subset was negatively correlated with both CD79B and HLA-dra levels. Overall, the presence of immune cells was negatively correlated with the levels four plasma proteins (CD79B, AGER, CAPS and HLA-dra). Specifically, the presence of five immune cell phenotypes (HLA-DR+CD33^-^ HLA DR+, HLA-DR+CD33brCD14dim, HLA-DR+ DCs, HLA-DR+ myeloid DCs and HLA-DR+ plasmacytoid DCs) were negatively correlated with the levels of two plasma proteins (CAPS, HLA-dra), the presence of four immune cell phenotypes (HLA-DR+CD33^-^ HLA DR+,HLA-DR+ DC, HLA-DR+ myeloid DCs and HLA-DR+ plasmacytoid DCs) were negatively correlated with AGER levels, and the presence of one immune cell phenotype (HLA-DR+CD33brCD14dim) was negatively correlated with CD79B levels (**Figure 4**). When plasma proteins were analyzed as exposures and immune cells as outcomes, the MR analysis revealed an inverse causal relationship between MICB levels and the presence of four immune cell phenotypes (HLA-DR+CD33^-^, HLA-DR+CD33brCD14dim, HLA-DR+ myeloid DCs, and HLA-DR+ plasmacytoid DCs) (**Figure 5**). Conversely, the MR analysis in which immune cells were treated as exposures and plasma proteins as outcomes revealed no causal effects of these phenotypes on MICB levels. These MR results suggest that MICB attenuates DPN via its inverse causal effect on specific immune cell phenotypes. Additionally, the level of HLA-DRA was inversely associated with the presence of two immune cell subtypes (HLA-DR+CD33brCD14dim and HLA-DR+ myeloid DCs). The MR analysis revealed no causal relationship between the levels of three plasma proteins (CD79B, AGER, and CAPS) and the presence of the five immune cell phenotypes examined (all P > 0.05). Conversely, PRKCG level was positively associated with the presence of HLA-DR+ plasmacytoid DCs (P < 0.05). In our final analysis, we conducted a mediation analysis to elucidate the causal relationships between immune cell phenotypes and DPN mediated by plasma proteins. We discovered that plasma proteins mediated the relationship between 5 immune cell phenotypes and DPN (P < 0.05). Notably, the effects of all immune cells were mediated by two distinct plasma proteins (CAPS and HLA-dra) (**Table 1**).

**Table 1:** Mediation analysis of the impact of circulating immune cells on DPN using a two-step Mendelian randomization framework.

Immune cell	Plasma proteins	Outcome	β (95%CI)	β1 (95%CI)	β2 (95%CI)	Mediation effect (95%CI)	Mediation proportion (95%CI)	P value
HLA DR on CD33− HLA HR+	AGER	DPN	0.42(0.23, 0.62)	-0.10(-0.15, -0.05)	-0.25(-0.36, -0.13)	0.02 (0.01, 0.04)	6.15% (0.00, 12%)	0.00
	CAPS	DPN	0.42(0.23, 0.62)	-0.17(-0.25, -0.08)	-1.54(-2.06, -1.01)	0.26(0.12, 0.42)	60.88% (28.30%, 98.28%)	<0.001
	HLA-dra	DPN	0.42(0.23, 0.62)	-0.40(-0.67, -0.13)	-0.64(-0.89, -0.39)	0.26(0.06, 0.49)	60.92% (14.65%, 114.18%)	0.002
HLA DR on CD33br HLA DR+ CD14dim	CD79B	DPN	0.24(0.11,0.37)	-0.05(-0.10, -0.01)	-2.30(-3.13,-1.47)	0.13(0.04, 0.25)	55.14% (18.16%-103.48%)	<0.001
	CAPS	DPN	0.24(0.11,0.37)	-0.08(-0.16,0.01)	-1.54(-2.06, -1.01)	0.13(0.03, 0.26)	53.55%(11.90%-108.56%)	0.001
	HLA-dra	DPN	0.24(0.11,0.37)	-0.46(-0.80, -0.12)	-0.64(-0.89, -0.39)	0.30(0.09, 0.57)	124.22%(37.93%, 238.27%)	<0.001
HLA DR on DC	AGER	DPN	0.45(0.28, 0.82)	-0.08(-0.15,-0.003)	-0.25(-0.36, -0.13)	0.02(0.004, 0.04)	4.47% (0.90%, 9.64%)	0.007
	CAPS	DPN	0.45(0.28, 0.82)	-0.15(-0.23, -0.07)	-1.54(-2.06, -1.01)	0.23(0.10, 0.40)	52.26% (22.22%, 90.02%)	<0.001
	HLA-dra	DPN	0.45(0.28, 0.82)	-0.70(-0.87, -0.54)	-0.64(-0.89, -0.39)	0.46(0.25, 0.71)	101.36% (55.27%, 157.41%)	<0.0001
HLA DR on myeloid DC	AGER	DPN	0.98(0.70, 1.27)	-0.30(-0.45,-0.14)	-0.63(-1.02, -0.24)	0.19(0.064, 0.35)	19.30% (6.53%, 35.87%)	<0.001
	CAPS	DPN	0.48(0.24,0.73)	-0.16(-0.28,-0.04)	-0.25(-0.36, -0.13)	0.24(0.09, 0.44)	50.66% (19.00%, 91.49%)	<0.001
	HLA-dra	DPN	0.48(0.24,0.73)	-0.45(-0.72,-0.18)	-0.64(-0.89, -0.39)	0.29 (0.12, 0.51)	60.36% (25.45%, 105.38%)	<0.001
HLA DR on plasmacytoid DC	AGER	DPN	0.41(0.28, 0.55)	-0.09(-0.16, -0.01)	-0.25(-0.36, -0.13)	0.02(0.003, 0.05)	5.42% (0.93%, 11.93%)	0.008
	CAPS	DPN	0.41(0.28, 0.55)	-0.15(-0.23, -0.08)	-1.54(-2.06, -1.01)	0.24(0.11, 0.41)	57.76% (26.89%, 99.42%)	<0.001
	HLA-dra	DPN	0.41(0.28, 0.55)	-0.42(-0.59, -0.25)	-0.64(-0.89, -0.39)	0.27 (0.13, 0.47)	66.33% (32.11%, 112.41%)	<0.001

### Validating the role of conventional dendritic cells at the single-cell level

**Figure 6:**
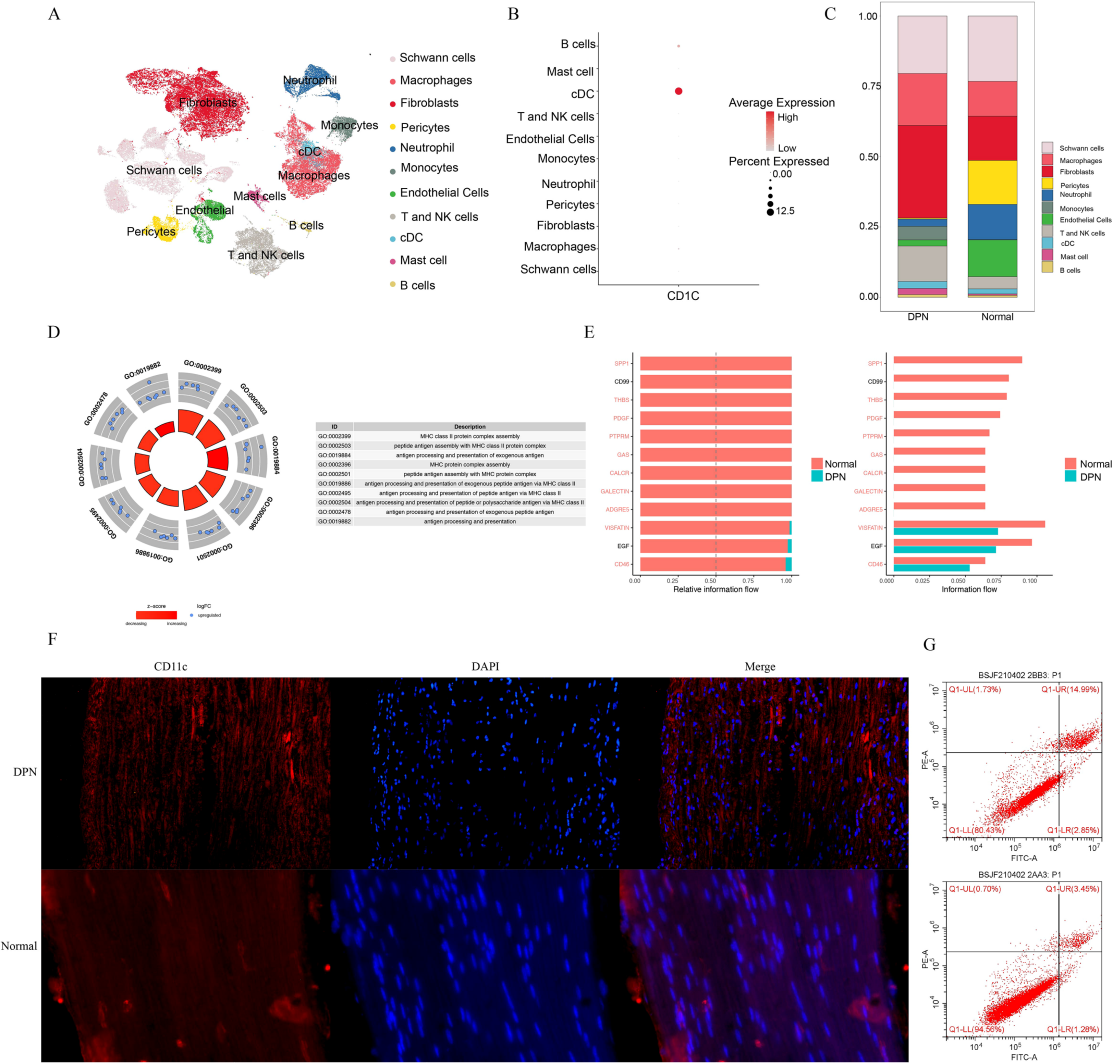
Single-Cell Profiling of human peripheral nerves identifies cDC enrichment in DPN. **(A)**Uniform manifold approximation and projection (UMAP) clustering of sural nerve samples from 4 DPN patients and 3 TLA controls. **(B)** cDCs frequency showed a significant increase in DPN nerves versus control nerves. **(C)** Gene Ontology enrichment analysis indicated that cDCs are involved in MHC class II complex assembly, peptide antigen loading, and exogenous antigen processing and presentation. **(D)** The cell–cell communication analysis revealed reduced neuroreparative signaling activity between cDCs and Schwann cells. **(E)** Increased DC infiltration in DPN nerves confirmed by immunofluorescence. **(F)** Elevated dendritic cells in DPN patient blood were validated by flow cytometry.

MR identified five immune cell types causally linked to DPN. Given that three types of cells are conventional dendritic cells (cDCs), we performed single-cell RNA sequencing to investigate their roles in DPN pathogenesis. After quality control, 106,729 cells (31,876 normal; 74,853 DPN) were clustered into 11 populations: Schwann cells, macrophages, fibroblasts, pericytes, neutrophils, monocytes, endothelial cells, T/NK cells, mast cells, B cells, and CD11C^+^ cDCs (**Figure 6A**). The cDC frequency exhibited a statistically significant twofold increase in the nerves of subjects with DPN (0.02%) than in those of healthy controls (0.01%; P < 0.05) (**Figure 6B**). The Gene Ontology enrichment analysis suggested that cDCs are involved in MHC class II complex assembly, peptide antigen loading, and exogenous antigen presentation (**Figure 6C**). The cell–cell communication analysis revealed reduced neuroreparative signaling (e.g., PDGFR and EGFR signaling) activity between conventional dendritic cells (cDCs) and Schwann cells in individuals with DPN, indicating diminished cDC-mediated trophic support for Schwann cells (**Figure 6D**).This finding was corroborated by the observation of elevated cDC infiltration in DPN nerves by immunofluorescence staining and flow cytometry, confirming the increased circulating cDC frequency in the plasma of DPN patients (**Figures 6E, F**).

## Discussion

In this study, we employed MR models integrated with GWAS data to systematically investigate causal relationships among circulating immune cells, plasma proteins, and DPN. Our analyses revealed five distinct immune cell phenotypes—primarily HLA-DR^+^ dendritic cell subsets—with significant causal effects on an increased DPN risk, alongside six plasma proteins (MICB, HLA-DRA, CAPS, CD79B, AGER, and PRKCG), which exhibited protective causal effects on DPN. We established that pathogenic HLA-DR^+^ dendritic cell subsets increase the DPN risk by suppressing protective mediators—specifically, reducing CAPS and HLA-DRA expression, which mediate 26.3% of immune-induced neurotoxicity (P<0.001). Conversely, MICB conferred protection not only directly but also by inhibiting causal immune phenotypes. Crucially, single-cell validation resolved the spatial dynamics of this network: cDC infiltration in DPN nerves impaired Schwann cell reparative signaling, mechanistically substantiating MR-derived causal pathways.

Emerging evidence consistently highlights the role of immune-metabolic dysregulation across various neuropathic conditions, including chemotherapy-induced peripheral neuropathy (CIPN) and diabetic neuropathy. Meregalli et al. comprehensively reviewed blood molecular biomarkers—such as neurotrophins, microRNAs, neurofilaments, and metabolites—in CIPN, emphasizing their potential in predicting axonal injury and neuroinflammation (11). Similarly, Bonomo outlined the clinical and mechanistic complexity of diabetic neuropathy, noting inflammatory pathways and immune cell involvement as key contributors (12). In this study, we revealed HLA-DR^+^ dendritic cells as primary causal drivers of the DPN risk, with specific myeloid subsets contributing secondarily. DCs serve as the paramount sentinels of the immune system, orchestrating immune responses through the integration of signals across immune compartments (13). Multiple studies have shown that the recruitment of dendritic cells to islets can cause an inflammatory response during early diabetes, including the apoptosis of islet cells, viral pathogen-induced cross-presentation of endogenous peptides, or superantigen-triggered immune responses during early diabetes (14, 15). In diabetic retinopathy, which is a component of DPN, animal studies have shown that chronic hyperglycemia may be recognized by DCs, triggering cellular apoptosis and consequently reducing the density of sensory nerve fibers and their terminals (16). Our single-cell analysis revealed that cDC infiltration in DPN nerves disrupts Schwann cell reparative signaling, mechanistically bridging MR-identified causality with human tissue pathology. Moreover, HLA-DR^+^ immune cells drive DPN pathogenesis, whereas the plasma HLA-DRA level has a protective effect (β=-0.34, p<0.001). This finding aligns with emerging evidence of compartmentalized HLA functions: membrane-bound HLA-DR potentiates antigen presentation to autoreactive T cells in nerves (17), whereas circulating HLA-DR dampens neuroinflammation through competitive receptor blockade (18, 19). In this study, increased plasma HLA-DRA levels may represent an adaptive response countering DC-mediated nerve damage, although its therapeutic potential requires further validation. Furthermore, our MR analysis identified the HLA-DR^+^CD33^-^ and HLA-DR^+^CD33^br^CD14^dim^ immune phenotypes as causal risk factors for DPN. Notably, these phenotypes involve myeloid-derived suppressor cells (MDSCs), which are involved in MDSC activation in DPN pathogenesis, a finding that is consistent with the established role of MDSCs in microangiopathy associated with diabetic retinopathy (DR) (20). Under physiological conditions, myeloid precursors undergo terminal differentiation into dendritic cells, macrophages, and granulocytes. In pathological states characterized by chronic inflammation, such as diabetes, this differentiation program is arrested, leading to the aberrant accumulation of immunosuppressive MDSCs (21). MDSCs comprise two main subpopulations: granulocytic MDSCs (G-MDSCs; also termed polymorphonuclear MDSCs, PMN-MDSCs), which exhibit a neutrophil-like morphology, and monocytic MDSCs (M-MDSCs), which morphologically resemble monocytes (22). Additionally, a more immature subpopulation exists, termed early-stage MDSCs (E-MDSCs), which lacks specific granulocytic or monocytic markers (23). We observed significantly elevated peripheral MDSC levels in DPN patients, driven primarily by E-MDSCs (CD14^-^CD33^+^HLA-DR^-^). This finding aligns with reports of increased CD14^-^ MDSCs in individuals with diabetic kidney disease (21). Collectively, these findings establish aberrant MDSC expansion driven by chronic inflammation-mediated arrest of myeloid differentiation as a novel pathogenic mechanism of DPN, providing a conceptual link to microangiopathy in diabetic complications and highlighting E-MDSCs as potential therapeutic targets.

Our analyses also revealed that six plasma proteins exhibited protective causal associations with a reduced DPN risk. Furthermore, the GO analysis showed that these proteins are involved in the biological processes of leukocyte activation and cognition. MICB encodes a stress-induced, glycosylated NKG2D ligand that triggers cytolysis in NKG2D^+^ lymphocytes (natural killer cells, CD8+ T cells) (24). Some MR studies have documented its association with diabetes-related complications (25, 26). However, prior studies have not established an association between MICB and DPN. Here, we demonstrate that plasma MICB confers protection against DPN both directly and through the suppression of causal immune phenotypes. Mechanistically, MICB-NKG2D engagement activates STAT3 signaling to promote MDSC induction (27), supporting our finding of an inverse association between plasma MICB levels and the presence of pathogenic E-MDSCs in patients with DPN. CAPS encodes a calcium-binding protein involved in regulating ion transport (28). In this study, CAPS levels were inversely associated with diabetic peripheral neuropathy (DPN), and the presence of five immune cell subtypes were negatively correlated with CAPS level. The protein localizes to the cytosol and is widely expressed in various tissues, including endocrine glands (thyroid, pancreas, adrenal, and pituitary) and epithelial linings (respiratory, digestive, and genitourinary) (29). However, research on the relationship between the CAPS and immune cell function remains limited, and its role in DPN pathogenesis requires further exploration. Our research also confirmed that the presence of four immune cell subtypes was inversely correlated with AGER levels. Research on the role of AGER, which is a specific receptor for advanced glycosylation end products (AGEs), in diabetes is extensive. When AGEs bind to AGER, they activate proinflammatory pathways. AGER exists in two primary isoforms: membrane-bound RAGE (mRAGE) and soluble AGER (sRAGE). Plasma AGER is the soluble form, and competitively binds to irculating advanced glycosylation end products (AGEs). This binding blocks the interaction of AGEs with the transmembrane receptor RAGE (mRAGE), thereby inhibiting the downstream proinflammatory signaling pathways mediated by reactive oxygen species (ROS) and nuclear factor kappa B (NF-κB) (30). Yuan S demonstrated that AGER was more likely to be associated with acute and chronic microvascular complications than with macrovascular complications in patients with diabetes (7), which is supported by our findings. CD79B is a core component of the B-cell antigen receptor (BCR) complex and plays an essential role in B-cell signal transduction. Genetic knockout of CD79B in mice significantly impairs the antigen response capacity of B cells, confirming the critical functions of CD79B in BCR signaling pathways (31). In individuals with diabetes, B cells promote autoimmunity through multiple mechanisms, including autoantibody production, antigen presentation, and proinflammatory cytokine secretion (32). Nevertheless, the role of plasma CD79B in diabetic complications remains underexplored. Our study establishes a significant inverse association between plasma CD79B levels and diabetic peripheral neuropathy (DPN). In this study, none of the five DPN-associated immune cell subtypes correlated with plasma PRKCG levels; however, a significant negative association was observed between PRKCG levels and DPN. As a calcium-dependent protein kinase C gamma isoform, PRKCG potentiates interneuron excitability in response to neural damage, which attenuates neuropathic pain through the modulation of synaptic transmission (33). In summary, these six plasma proteins orchestrate a concerted defense against DPN pathogenesis, where their complementary mechanisms—from MICB-mediated immune suppression to AGER-dependent AGE neutralization and PRKCG/CAPS neuroprotection—converge into a multitarget therapeutic framework for diabetic neuropathy intervention.

While our integrative MR and single-cell approach established a novel causal neuroimmune network in DPN, several limitations inherent to the study design and available data must be acknowledged. As a static causal inference method, MR captures lifelong genetic effects but cannot model the dynamic temporal interplay between immune cells and plasma proteins across different stages of DPN initiation, progression, and response to therapy. Although our scRNA-seq analysis provided crucial spatial and cellular context by revealing cDC infiltration and disrupted Schwann cell signaling in diseased nerves, it represents a single time point. Future longitudinal multi-omics studies in serial human samples or staged preclinical models are essential to map the evolution of this network. While MR adeptly identifies whether an exposure causally influences DPN, it does not reveal the underlying molecular mechanisms. For instance, we infer but do not experimentally prove how HLA-DR^+^ dendritic cells suppress CAPS/HLA-DRA expression, or through which precise signaling pathways MICB or soluble AGER exert protection. These remain testable hypotheses, and targeted experimental validation using co-culture systems, genetic perturbations, and animal models is a critical next step. Although MR significantly reduces confounding by leveraging genetic instruments, residual bias from unmeasured clinical factors (precise diabetes duration, glycemic variability) cannot be fully excluded. While our sensitivity analyses (MR-Egger, weighted median, leave-one-out) support the robustness of our main findings, future multivariable MR analyses incorporating genetic proxies for these clinical variables would be valuable. Finally, this study was conducted primarily at the population level using European-ancestry GWAS data. We did not perform stratified analyses by key demographic factors such as age and sex, which are known to modulate immune responses and neuropathy risk. This was primarily due to the current lack of large-scale, publicly available GWAS summary statistics for DPN stratified by these variables. Future research should prioritize such stratified causal inference and include diverse populations to assess the generalizability and potential heterogeneity of the identified immune-plasma protein-DPN pathways.

Overall, our integrative Mendelian randomization study establishes a causal neuroimmune network that drives diabetic peripheral neuropathy (DPN), in which pathogenic HLA-DR^+^ dendritic cell subsets reduce the DPN risk by suppressing the expression of protective plasma proteins, notably CAPS and HLA-DRA. Conversely, we identified six plasma proteins as novel mediators with protective effects on DPN, with MICB conferring dual protection through direct effects and the suppression of causal immune phenotypes. Single-cell validation mechanistically resolved cDC infiltration in DPN nerves and the disruption of Schwann cell reparative signaling, bridging genetic causality with tissue pathology. These findings position HLA-DR^+^ immune cells as therapeutic targets while proposing plasma proteins as both biomarkers and multitarget candidates for intervention in DPN.

## Data Availability

The data presented in the study are deposited in the GEO repository (https://www.ncbi.nlm.nih.gov/geo/), accession number GSE266026.

## Supplementary material

Supplementary Table 1 No heterogeneity was observed in the causal effects of immune cells on DPN.

Supplementary Table 2 All six causal proteins showed consistent directional effects across MR methods (MR-Egger, weighted median, weighted mode) and IVW analyses.

Supplementary Table 3 MR analysis using the six plasma proteins as exposures and DPN as outcome showed no horizontal pleiotropy in MR-PRESSO testing (p > 0.05).

Supplementary Figure 1 Leave-one-out sensitivity analysis confirmed robust causal effects of immune cells on DPN, with overall estimates remaining stable after iterative removal of individual instrumental variables.

Supplementary Figure 2 Reverse MR analysis indicated no causal effect of DPN on immune cells.
