# Supplementary figures and images for: Immunoproteomic mediators of diabetic peripheral neuropathy: causal insights from Mendelian randomization and single-cell validation
Source: Front Immunol. 2026 Jan 29;17:1681223. doi: 10.3389/fimmu.2026.1681223 (PMC12894356; doi:10.3389/fimmu.2026.1681223)

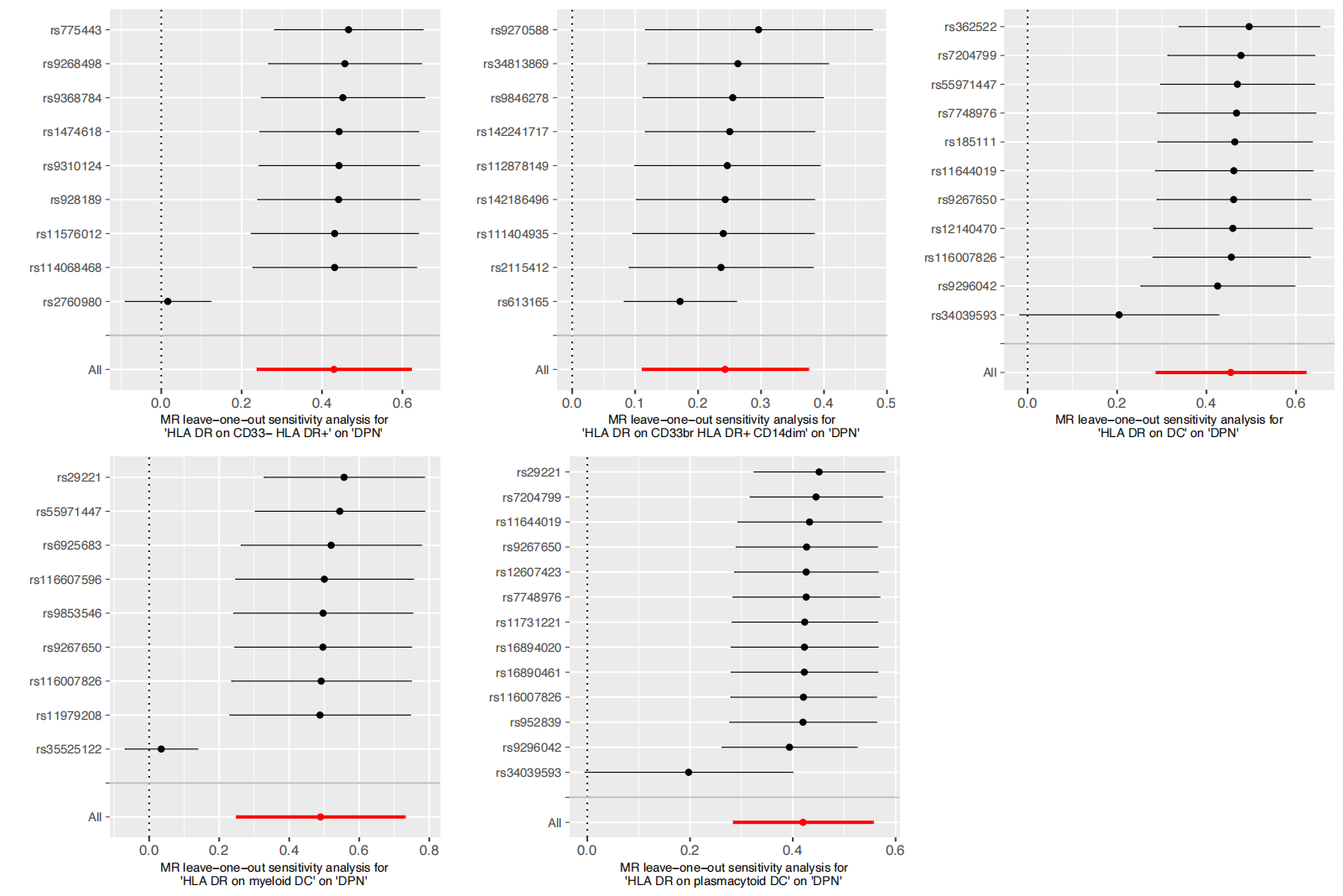

Supplement: Supplementary Figure 1 — Leave-one-out sensitivity analysis confirmed robust causal effects of immune cells on DPN, with overall estimates remaining stable after iterative removal of individual instrumental variables. [file Image1.tif]

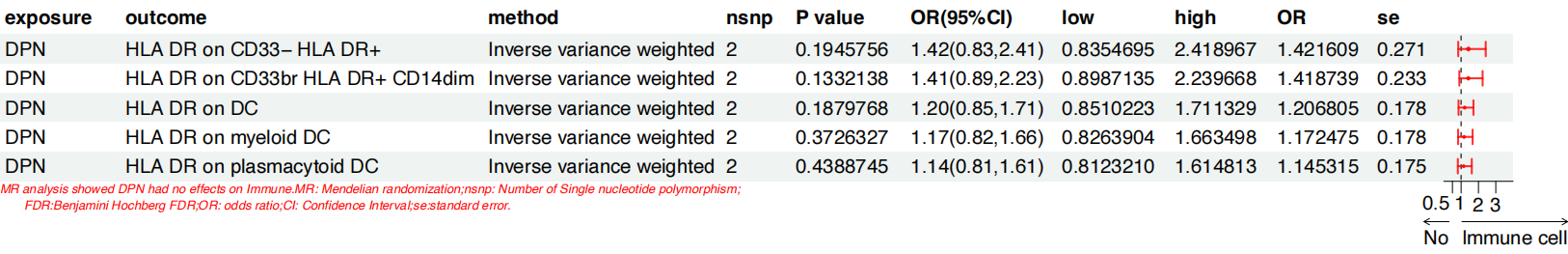

Supplement: Supplementary Figure 2 — Reverse MR analysis indicated no causal effect of DPN on immune cells. [file Image2.tif]
